# A Computational Study of Potential miRNA-Disease Association Inference Based on Ensemble Learning and Kernel Ridge Regression

**DOI:** 10.3389/fbioe.2020.00040

**Published:** 2020-02-06

**Authors:** Li-Hong Peng, Li-Qian Zhou, Xing Chen, Xue Piao

**Affiliations:** ^1^School of Computer Science, Hunan University of Technology, Zhuzhou, China; ^2^School of Information and Control Engineering, China University of Mining and Technology, Xuzhou, China; ^3^School of Medical Informatics, Xuzhou Medical University, Xuzhou, China

**Keywords:** miRNA, disease, association prediction, ensemble, kernel ridge regression

## Abstract

As increasing experimental studies have shown that microRNAs (miRNAs) are closely related to multiple biological processes and the prevention, diagnosis and treatment of human diseases, a growing number of researchers are focusing on the identification of associations between miRNAs and diseases. Identifying such associations purely via experiments is costly and demanding, which prompts researchers to develop computational methods to complement the experiments. In this paper, a novel prediction model named Ensemble of Kernel Ridge Regression based MiRNA-Disease Association prediction (EKRRMDA) was developed. EKRRMDA obtained features of miRNAs and diseases by integrating the disease semantic similarity, the miRNA functional similarity and the Gaussian interaction profile kernel similarity for diseases and miRNAs. Under the computational framework that utilized ensemble learning and feature dimensionality reduction, multiple base classifiers that combined two Kernel Ridge Regression classifiers from the miRNA side and disease side, respectively, were obtained based on random selection of features. Then average strategy for these base classifiers was adopted to obtain final association scores of miRNA-disease pairs. In the global and local leave-one-out cross validation, EKRRMDA attained the AUCs of 0.9314 and 0.8618, respectively. Moreover, the model’s average AUC with standard deviation in 5-fold cross validation was 0.9275 ± 0.0008. In addition, we implemented three different types of case studies on predicting miRNAs associated with five important diseases. As a result, there were 90% (Esophageal Neoplasms), 86% (Kidney Neoplasms), 86% (Lymphoma), 98% (Lung Neoplasms), and 96% (Breast Neoplasms) of the top 50 predicted miRNAs verified to have associations with these diseases.

## Introduction

MicroRNAs (miRNAs), known as the member of short non-coding RNA family, are found in eukaryotic organisms including viruses, plants and animals. They negatively regulate the expression of messenger RNA (mRNA) and the protein translation of their target genes (Bartel, 2004). In addition, miRNAs could also play a role of positive regulators demonstrated in some previous studies (Jopling et al., 2005; Vasudevan et al., 2007). Under normal physiological conditions, miRNAs function in feedback mechanisms by safeguarding key biological processes including cell proliferation, differentiation and apoptosis (Bruce et al., 2015; Reddy, 2015). Many researchers have studied and validated the dysregulation of miRNA expression in various disease conditions (Esquelakerscher and Slack, 2006; Latronico et al., 2007; Lynam-Lennon et al., 2009; Meola et al., 2009; Wilczynska and Bushell, 2015). For example, Jeong et al. (2011) clarified that let-7a was under-expressed in the blood, cells and tissues of non-small cell lung cancer (NSCLC) patients compared to normal controls; and that the possibility of using let-7a as a serologic marker for lung cancer detection needed further study. Liang et al. (2012) reported that 66 miRNAs were differentially expressed in denatured dermis compared with those in normal skin; and the most significantly up-regulated miRNA was miR-663, while miR-203 was the most significantly down-regulated one. They further pointed out that identifying different miRNA expressions could enhance the understanding the mechanisms behind the functional recovery of the denatured dermis. Besides, miR-23/27/24 cluster has been shown by experiments to be involved in angiogenesis and endothelial apoptosis in cardiac ischemia and retinal vascular development (Bang et al., 2012). Hence, it is necessary and urgent to discover more miRNA-disease associations, contributing the prevention, diagnosis and treatment of complex human diseases. Nevertheless, it costs much time and money to discover true disease-related miRNAs from a mass of candidates by traditional biological experiments (Calin and Croce, 2006). Nowadays, many computational models of predicting miRNA-disease associations were developed based on some biological datasets, which could be used as an important complement to biological experiments (Chen et al., 2015).

Considering the hypothesis that functionally similar miRNAs tend to be related to similar diseases (Wang et al., 2010; Chen et al., 2019), some scoring function-based methods have been established to reveal new miRNA-disease associations. For example, Jiang et al. (2010) designed an initial computational model to infer potential disease-associated miRNAs. The approach integrated miRNA functional similarity network, the disease phenotype similarity network and the known miRNA-disease association network to prioritize the entire human miRNAome for the investigated disease with a cumulative hypergeometric distribution. But, the model failed to achieve excellent results because only neighbor information of miRNAs was used in the model. Moreover, Xuan et al. (2013) presented a prediction method of Human Disease-MiRNA association Prediction (HDMP) based on weighted *k* most similar neighbors of unlabeled miRNAs that have no known associations with disease *d*. However, HDMP could not predict related miRNAs for new diseases having no known association information. Additionally, the model also only used local network similarity. Furthermore, Chen et al. (2016b) proposed a new computational approach of Within and Between Score for MiRNA-Disease Association prediction (WBSMDA), which integrated known miRNA-disease associations, the miRNA functional similarity, the disease semantic similarity and the Gaussian interaction profile (GIP) kernel similarity for diseases and miRNAs. The authors first defined Within-Score and Between-Score in the side of miRNAs and diseases, respectively. Then the association score of the investigated miRNA-disease pair could be obtained by combining the corresponding Within-Score and Between-Score. However, WBSMDA did not exhibit outstanding performance because it was difficult to integrate Within-Score and Between-Score in reasonable way. Pasquier and Gardes (2016) introduced the MiRAI model that concatenated multiple miRNA-related association networks. Then dimensionality reduction technique was conducted for the combined network with Singular Value Decomposition (SVD). The final miRNA-disease association scores were attained by calculating cosine similarity between miRNA vectors in the miRNA space and disease vectors in the disease space.

Moreover, some network-based models were put forward. For example, Chen et al. (2012) proposed the model of Random Walk with Restart for MiRNA-Disease Association prediction (RWRMDA). The model was the first to adopt global network similarity measures and carried out random walk on the miRNA functional similarity network. However, the model also had the important limitation that it was not applicable to new diseases having no known association information. Another model named MIDP was presented by Xuan et al. (2015) for miRNA-disease association prediction. The model also performed random walk on the miRNA functional similarity network. To predict miRNA-disease associations for new diseases having no association information, the miRNA-disease bilayer network was built and MIDP could implement walk on this network. Furthermore, Chen et al. (2016c) presented the computational method of Heterogeneous Graph Inference for MiRNA-Disease Association prediction (HGIMDA), the inputs of which was same as WBSMDA. HGIMDA implemented an iterative process on the constructed heterogeneous graph to predict potential miRNA-disease associations. The model’s performance was better than many previous models. In order to obtain better performance, Chen et al. (2018f) constructed Matrix Decomposition and Heterogeneous Graph Inference (MDHGI) to infer disease-related miRNAs. Before implementing heterogeneous graph inference similar to HGIMDA, the authors employed matrix factorization for miRNA-disease adjacent matrix to remove redundant information. Gu et al. (Gu et al., 2016) proposed another method named Network Consistency Projection for miRNA-Disease Associations (NCPMDA). Firstly, the authors constructed similarity network for miRNAs and diseases, respectively, by integrating multiple heterogeneous biological data. Secondly, the authors performed network consistency projection from the miRNA (disease) similarity network to the miRNA-disease association network, respectively. Lastly, scores of both network consistency projections were combined as the final miRNA-disease association score. Yu et al. (2017) introduced a prediction method named MaxFlow for miRNA-disease association prediction. In this method, miRNAome-phenome network was constructed by combining multiple heterogeneous network. For an investigated disease, the authors adopted push-relabel maximum flow algorithm to compute the maximum information flow from the source node over all links to sink node, and used the flow quantity leaving a miRNA node as the association score between the investigated disease and miRNA. In addition, You et al. (2017) developed the model of Path-Based MiRNA-Disease Association prediction (PBMDA). This model constructed a heterogeneous graph, adopted depth-first search algorithm to traverse all paths between a miRNA node and a disease node, and used the product of all the edges’ weights as path score in each path. The miRNA-disease association score could be obtained by summing all path scores between the miRNA node and disease node. In this model, distance-decay function was used for further weakening the score contribution of longer path. In addition, Chen et al. (2018e) presented the method of Bipartite Network Projection for MiRNA–Disease Association prediction (BNPMDA), which built bias ratings for miRNAs (diseases) by using agglomerative hierarchical clustering and improved traditional bipartite network recommendation. Although BNPMDA obtained better prediction accuracy than many previous models, it is not applicable for new diseases without known related miRNAs.

In fact, many previous studies were carried out with the addition of other association networks. For instance, Shi et al. (2013) developed a method to exploit associations between miRNAs and diseases by implementing a random walk analysis that focused on the functional links between the miRNA targets in the protein-protein interaction (PPI) network and the disease genes. Because of involving the PPI network, the model’s prediction performance was improved and better than that of many previous models. In addition, Mørk et al. (2014) proposed a model named miRPD that integrated the protein-disease relations and the miRNA-protein interactions, and could effectively predict new associations between miRNAs and diseases.

With the development of machine learning algorithms, many researchers have begun to use this technology to solve various biological problems, such as prediction of drug-target interactions (Chen et al., 2016d), synergistic drug combinations (Chen et al., 2016a), disease related long non-coding RNAs (Chen et al., 2017c), miRNA-small molecule associations (Chen et al., 2018b), genome-wide features (Xu et al., 2018) and functional impact of variants (Milanese et al., 2019; Rojano et al., 2019; Xu et al., 2019). Of course, some machine learning models were developed for predicting potential associations between miRNAs and diseases (Chen et al., 2018a,c,g; Wang et al., 2019). For example, the model of Restricted Boltzmann Machine for Multiple types of MiRNA-Disease Association prediction (RBMMMDA) was proposed by Chen et al. (2015). It was worth noting that RBMMMDA could reveal association types for predicted miRNA-disease associations, which was different from other prediction models. However, RBMMMDA only took the advantage of the information of known multiple types of miRNA-disease associations, which hindered it from achieving an excellent performance. Xu et al. (2011) introduced a model based on a heterogeneous MiRNA-Target Dysregulated Network (MTDN). Features were extracted based on the network and a support vector machine (SVM) classifier was constructed to differentiate positive miRNA–disease associations from negative associations. However, the performance of this method was significantly influenced by an inaccurate selection of negative samples. In addition, another computational model of Regularized Least Squares for MiRNA-Disease Association prediction (RLSMDA) was proposed by Chen and Yan (2014). RLSMDA could implemented prediction for new miRNAs and new diseases without known association information. Moreover, negative samples were not needed in the model because RLSMDA was based on a semi-supervised learning-based model. However, the selection of parameter values limited the performance of RLSMDA. For a further improvement, Chen et al. (2017a) developed a computational model based on Super-Disease and MiRNA for potential MiRNA–Disease Association prediction (SDMMDA). In order to obtain more accurate miRNA (disease) similarity measures, the concepts of “super-miRNA” and “super-disease” were introduced into the model. Furthermore, Chen et al. (2017b) constructed a computational method of Ranking-based K-Nearest-Neighbors (KNN) for MiRNA-Disease Association prediction (RKNNMDA). The KNN of miRNA and diseases were obtained from their similarity scores. Then these KNN were reranked with a Support Vector Machine (SVM) ranking model, and finally, voting was weighted and final ranking of all possible miRNA-disease pairs was obtained. However, RKNNMDA did not show excellent prediction performance with an AUC (Area Under the ROC Curve) of 0.8221 in leave-one-out cross validation (LOOCV). Li et al. (2017) proposed the method of Matrix Completion for MiRNA-Disease Association prediction (MCMDA). In the model, the adjacency matrix of known miRNA-disease associations could be updated based on matrix completion technology, without requiring negative associations as needed by several previous models. Furthermore, Chen et al. (2018d) proposed another matrix completion-based method named Inductive Matrix Completion for MiRNA-Disease Association prediction (IMCMDA), which utilized miRNA (disease) similarity as features to train the model and complete the missing miRNA-disease associations. Furthermore, Chen and Huang (2017) developed the novel prediction method of Laplacian Regularized Sparse Subspace Learning for MiRNA-Disease Association prediction (LRSSLMDA). First, the model extracted feature profiles for miRNAs/diseases and formed graph Laplacian matrices. Second, a common subspace for the miRNA/disease feature profiles, a *L*_1_-norm constraint and Laplacian regularization terms were joint to construct the objective function from miRNA and disease perspective, respectively. Third, the projection matrices in objective functions were iteratively updated and we obtained the final project matrix. Fourth, the association score between the miRNA and disease was computed using final projection matrix and feature profiles from miRNA and disease perspective respectively, and then the average of these two scores was the final prediction result. In addition, Zhao et al. (2019) further proposed the model named Adaptive Boosting for MiRNA-Disease Association prediction (ABMDA). In order to balance positive samples and negative samples, all unknown samples were divided into *k* clusters with *k*-means clustering and the same amount of negative samples were randomly selected from each cluster, and the number of total negative samples was almost equal to the positive. Then the authors integrated multiple weak classifiers (decision trees) to build a strong classifier based on corresponding weights for prediction.

As mentioned above, there were various limitations on previous prediction methods. Developing new and effective computational methods for potential miRNA-disease association prediction is in urgent need. Some computational methods have been proposed based on the assumption that functionally similar miRNAs tend to relate to similar diseases (Wang et al., 2010; Chen et al., 2019). Therefore, we considered using miRNA functional (disease semantic) similarity as miRNA (disease) features to develop a machine learning-based method for miRNA-disease association prediction. In addition, given that miRNA functional and disease semantic similarity was not complete, GIP kernel similarity for miRNAs and diseases could be utilized to supplement similarity information. Therefore, we obtained integrated miRNA (disease) similarity features and introduced a framework based on ensemble learning and feature dimensionality reduction to construct prediction model. In each base learning process, a feature subspace was firstly built by randomly choosing a set of integrated similarity features. Secondly, a dimensionality reduction method called Truncated Singular Value Decomposition (TSVD) was used to reduce the number of features in the feature subspace. Finally, we used Kernel Ridge Regression (KRR) to construct two classifiers in the miRNA space and the disease space, respectively, and they were integrated as the base classifier. The above base learning process was conducted repeatedly to yield many base classifiers based on random selection of features. The average of all the association scores from base classifiers was computed to obtain the final prediction results. This new model was named as Ensemble of Kernel Ridge Regression based on MiRNA-Disease Association prediction (EKRRMDA). In our work, EKRRMDA showed sound performance in cross validation and case studies. The AUCs were 0.9314 and 0.8618 in global and local LOOCV, respectively, and in 5-fold cross validation, the average and standard deviation of AUCs was 0.9275 ± 0.0008. Furthermore, we implemented three types of case studies: (1) using known miRNA-disease associations in the HMDD V2.0 database, (2) simulating new diseases that have no known association information by removing known associations for the investigated disease in the HMDD V2.0 database, and (3) using known miRNA-disease associations in HMDD V1.0 database to test model’s prediction performance in different datasets. The results showed that most miRNAs in top 50 predicted list were confirmed by experimental literature in case studies, which indicated that reliable prediction performance for the model.

## Materials and Methods

### Human miRNA-Disease Associations

In our study, the dataset of human miRNA-disease associations came from HMDD V2.0 database (Li et al., 2014), covering 5430 known miRNA-disease associations between 495 miRNAs and 383 diseases. An adjacency matrix *A* ∈ *R*^*m*×*n*^ (Variables *m* and *n* represent the number of miRNAs and diseases, respectively) was used to describe all information of miRNA-disease associations. If miRNA *m*_*i*_ was associated with disease *d*_*j*_, then *A*(*m*_*i*_, *d*_*j*_) was equal to 1, and 0 otherwise.

### miRNA Functional Similarity

Under the assumption that functionally similar miRNAs tend to be relate to semantically similar diseases, the method for calculating the miRNA functional similarity was proposed by Wang et al. (2010). MiRNA functional similarity could be obtained from http://www.cuilab.cn/files/images/cuilab/misim.zip and matrix *FS* was constructed to represent it.

### Disease Semantic Similarity Model 1

Disease semantic similarity were computed according to the methodology adopted in the literature (Xuan et al., 2013). At first, we obtained the relationship among various diseases from the Mesh database^1^ (Lipscomb, 2000; Wang et al., 2010). Then, we could use a graph *DAG*(*d*) = [*d*, *T*(*d*), *E*(*d*)] to describe disease *d*. Here, *T*(*d*) represented node set of all ancestor nodes of *d* and *d* itself, and *E*(*d*) was the corresponding direct edges set. Each disease *t* in *DAG*(*d*) has the contribution to the semantic value of disease *d* and we calculated the contribution as follows:

(1) $$\left\{ \begin{array}{c} D\,1_{d}\,\left(t\right)=1\,\mathrm{if}\,t=d\\ D\,1_{d}\,\left(t\right)=\max\left\{\Delta^{*}\,D\,1_{d}\,\left(t^{\prime }\right)|t^{\prime }\in \mathrm{children}\,\mathrm{of}\,d\right\}\,\mathrm{if}\,t\neq d \end{array}\right.\,$$

The semantic value of disease *d* could be defined as follows:

(2) $$D\,V\,1\,\left(d\right)=\sum_{t\in T\,\left(d\right)}D\,1_{d}\,\left(t\right)$$

where Δ was the semantic contribution decay factor. The above formula shows that diseases in different layers of *DAG*(*d*) had different contributions to the semantic value of disease *d.* For diseases that locate in different layers, their contributions to the semantic value of disease *d* decreased as distance between these diseases and disease *d* increased. Specially, it is easy to understand that we defined the contribution of disease *d* to semantic value of itself as 1. Based on the assumption that two diseases sharing a larger part of their DAGs have a larger similarity score, the semantic similarity score between disease *d*_*i*_ and *d*_*j*_ was defined as follows:

(3) $$S\,S\,1\,\left(d_{i},d_{j}\right)=\frac{\sum_{t\in T\,\left(d_{i}\right)\,⋂T\,\left(d_{j}\right)}\left(D\,1_{d_{i}}\,\left(t\right)+D\,1_{d_{j}}\,\left(t\right)\right)}{D\,V\,1\,\left(d_{i}\right)+D\,V\,1\,\left(d_{j}\right)}$$

where *SS*1 was disease semantic similarity matrix.

### Disease Semantic Similarity Model 2

The point that diseases in the same layers of *DAG*(*d*) have the same contribution to semantic value of disease *d* was adopted in disease semantic similarity model 1, however, it was not always reasonable. According to the literature (Xuan et al., 2013), another method of measuring disease semantic similarity was adopted. For example, if two diseases, *t*_1_ and *t*_2_, were located in the same layer of *DAG*(*d*) and disease *t*_1_ appeared in less DAGs than *t*_2_, disease *t*_1_ could be considered as a more specific disease and its contribution to semantic value of disease *d* should be higher than disease *t*_2_. So we defined the contribution of disease *t* in *DAG*(*d*) to the semantic value of disease *d* as follows:

(4) $$D\,2_{d}\,\left(t\right)=-\log\left[\,\frac{\mathrm{the}\,\mathrm{number}\,\mathrm{of}\,D\,A\,G\,s\,\mathrm{including}\,t}{\mathrm{the}\,\mathrm{number}\,\mathrm{of}\,\mathrm{diseases}}\right]$$

Similar to disease semantic similarity model 1, we could define the semantic similarity between disease *d*_*i*_ and *d*_*j*_ as follows:

(5) $$S\,S\,2\,\left(d_{i},d_{j}\right)=\frac{\sum_{t}\in T\,\left(d_{i}\right)\,⋂T\,\left(d_{j}\right)\,\left(D\,2_{d_{i}}\,\left(t\right)+D\,2_{d_{j}}\,\left(t\right)\right)}{D\,V\,2\,\left(d_{i}\right)+D\,V\,2\,\left(d_{j}\right)}$$

where *DV2*(*d*_*i*_) and *DV2*(*d*_*j*_) were semantic value of disease *d*_*i*_ and *d*_*j*_ in semantic similarity model 2, respectively.

### Gaussian Interaction Profile Kernel Similarity

Considering that not all miRNAs has functional similarity and so do diseases, the GIP kernel similarity for diseases and miRNAs were calculated according to van Laarhoven et al. (2011). By observing association information between miRNA *m*_*i*_ and each disease, binary vector *IV*(*m*_*i*_) was defined to represent the interaction profiles of miRNA *m*_*i*_. The GIP kernel similarity between miRNA *m*_*i*_ and *m*_*j*_ could be computed as follows:

(6) GM(mi,mj)=exp(-β|m|IV(mi)-IV(mj)||2)

(7) $$\beta_{m}=\beta_{m}^{\prime }/\left(\frac{1}{m}\,\sum_{i=1}^{m}{||I\,V\,\left(m_{i}\right)||}^{2}\right)$$

where adjustment coefficient β_*m*_ for the kernel bandwidth and $\beta_{m}^{\prime }$ was the original bandwidth. Similarly, disease GIP kernel similarity between disease *d*_*i*_ and *d*_*j*_ was computed as follows:

(8) $$G\,D\,\left(d_{i},d_{j}\right)=\exp\left(-\beta_{d}\,{||I\,V\,\left(d_{i}\right)-I\,V\,\left(d_{j}\right)||}^{2}\right)$$

(9) $$\beta_{d}=\beta_{d}^{\prime }/\left(\frac{1}{n}\,\sum_{i=1}^{n}{||I\,V\,\left(d_{i}\right)||}^{2}\right)$$

### Integrated Similarity for miRNAs and Diseases

According to the literature (Chen et al., 2016b), the integrated disease (miRNA) similarity was attained by combining the disease semantic (miRNA functional) similarity with GIP kernel similarity. Taking disease as an example, if there was semantic similarity between disease *d*_*i*_ and *d*_*j*_, then their integrated similarity was the mean of *SS1*(*d*_*i*_, *d*_*j*_) and *SS2*(*d*_*i*_, *d*_*j*_), otherwise we used GIP kernel similarity as the integrated disease similarity. The final integrated similarity between disease *d*_*i*_ and *d*_*j*_, was computed as follows:

(10) $$S\,D\,\left(d_{i},d_{j}\right)=\left\{ \begin{array}{c} \frac{S\,S\,1\,\left(d_{i},d_{j}\right)+S\,S\,2\,\left(d_{i},d_{j}\right)}{2}\,\,d_{i}\,\mathrm{and}\,d_{j}\,\mathrm{has}\,\mathrm{semantic}\\ G\,D\,\left(d_{i},d_{j}\right)\;\mathrm{similarityotherwise} \end{array}\right.$$

where *SD* represented integrated disease similarity matrix. For miRNAs, we defined the integrated miRNA integrated similarity between miRNA *m*_*i*_ and *m*_*j*_ as follows in the same way:

(11) $$S\,M\,\left(m_{i},m_{j}\right)=\left\{ \begin{array}{c} F\,S\,\left(m_{i},m_{j}\right)\;m_{i}\,\mathrm{and}\,m_{j}\,\mathrm{has}\,\mathrm{functional}\\ G\,M\,\left(m_{i},m_{j}\right)\,\mathrm{similarityotherwise} \end{array}\right.$$

where *SM* was denoted as integrated miRNA similarity matrix.

## Ekrrmda

**FIGURE 1:**
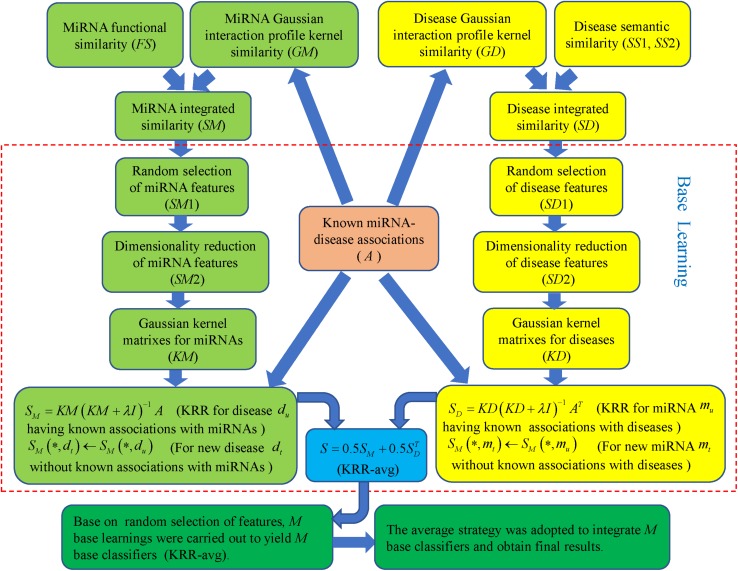
Flowchart of EKRRMDA to predict the potential miRNA-disease associations based on the known associations in HMDD V2.0 database.

Ensemble of Kernel Ridge Regression based MiRNA-Disease Association prediction was implemented by integrating known miRNA-disease association, miRNA functional similarity, disease semantic similarity and GIP kernel similarity for miRNAs and diseases. As formula (Wilczynska and Bushell, 2015) and (Jeong et al., 2011) showed, GIP kernel similarity was employed to supplement missing miRNA functional similarity and disease semantic similarity so that complete similarity information for miRNAs and diseases was obtained, respectively. Integrated similarity was used for miRNA and disease features that were the inputs to training model. Based on random selection of features, multiple base learnings were carried out to yield many base classifiers. Then average strategy was adopted to integrate these classifiers and get final prediction results [see Figure 1, motivated by important study from Ezzat et al. (2017)].

In each base learning, every row of similarity matrix *SM* (*SD*) is feature vector for the corresponding miRNA (disease). For example, *SM*(*m*_*i*_,^∗^) (*SD*(*d*_*i*_,^∗^)) represents feature vector miRNA *m*_*i*_ (disease *d*_*i*_), which reflects similarity information between miRNA *m*_*i*_ (disease *d*_*i*_) and other each miRNA (disease). During base learning, a set of features were firstly randomly selected for miRNAs and diseases. Here, we used parameter *r* (0 < *r* < 1) to determine number of selected features, which denotes proportion of selected features among all the features (*r* = 0.2 in our work). Here *m*_*s*_, (*m*_*s*_ = ⌊*r*×*m*⌋ represents the largest integrate that is not larger than *r*×*m*) miRNA features and *n*_*s*_ (*n*_*s*_ = ⌊*r*×*n*⌋) disease features were randomly sampled for each miRNA and disease, respectively. *SM*1 ∈ *R*^*m*×*m*_*s*_^ and *SD*1 ∈ *R*^*n*×*n*_*s*_^ denotes feature matrix of miRNAs and diseases after random feature selection, respectively.

Secondly, feature dimensionality reduction for miRNAs and diseases was further implemented to eliminate noises, redundancy, or irrelevant information and also improve computation efficiency. For method of dimensionality reduction, we chose TSVD that was developed from standard SVD (Xu, 1998). It took a given matrix *SM*1 and decomposed the matrix into *U* ∈ *R*^*m*×*k*_*m*_^, *S* ∈ *R*^*k*_*m*_×*k*_*m*_^ and *V* ∈ *R*^*m*_*s*_×*k*_*m*_^ such that *SM*1 = *USV*^*T*^, where *k*_*m*_ is truncation parameter and *S* was a diagonal matrix containing the largest *k*_*m*_ singular value of *SM*1. In our method, *k*_*m*_ = ⌊0.2×*m*_*s*_⌋ indicated that top 20% larger singular value of *SM*1 was saved and others were ignored. The reduced miRNA feature matrix could then be obtained as *SM2=US* which realized the compression of the column for the matrix *SM*1. Similarly, reduced disease feature matrix *SD*2 ∈ *R*^*n*×*k*_*d*_^ could be obtained from *SD*1 (*k*_*d*_ = ⌊0.2×*m*_*d*_⌋). Finally, *k*_*m*_ and *k*_*d*_ is 19 and 15, which represented the number of miRNAs features and disease features after dimensionality reduction.

Thirdly, based on the known miRNA-disease associations and the dimensionally-reduced features, we used KRR to build two classifiers in the miRNA space and disease space, respectively. KRR was kernel-based classifier, where Least Squares was used in the kernel-induced space (Vovk, 2013). At first, we computed Gaussian kernel matrices for the miRNA and disease from *SM*2 and *SD*2, respectively. For example, for a pair of miRNA *m*_*i*_ and *m*_*j*_, their Gaussian kernel similarity was computed as follows:

(12) $$K\,M\,\left(m_{i},m_{j}\right)=\exp\left(-\frac{||SM2\left(m_{i}{,}^{*}\right)-SM2\left(m_{j}{,}^{*}\right)|{|}^{2}}{k_{m}}\right)$$

where *SM*2(*m*_*i*_,^∗^) and *SM*2(*m*_*j*_,^∗^) are reduced feature vectors for miRNA *m*_*i*_ and *m*_*j*_, respectively. Analogically, Gaussian kernel similarity for the pair of disease *d*_*i*_ and *d*_*j*_ were computed as follows:

(13) $$K\,D\,\left(d_{i},d_{j}\right)=\exp\left(-\frac{||SD2\left(d_{i}{,}^{*}\right)-SD2\left(d_{j}{,}^{*}\right)|{|}^{2}}{k_{d}}\right)$$

Then two KRR classifiers could be established with Gaussian kernel matrixes in different spaces, respectively. Taking miRNA space as an example, for each the investigated disease, the KRR was trained using the miRNA kernel matrix *KM* and adjacency matrix *A* to obtain association score between every miRNA and the investigated disease. Considering all diseases in the manner of matrix, the least-squares solution could be obtained as follows:

(14) $$S_{M}=K\,M\,{\left(K\,M+\lambda \,I\right)}^{-1}\,A$$

where λ was a regularization parameter and we set its value as λ = 1, referring to the previous work (van Laarhoven et al., 2011), and *I* ∈ *R*^*m*×*m*^ is the identity matrix. However, above formula could not work for new diseases that had no known associations with miRNAs. we inferred association scores between the new disease and miRNAs according to integrated disease similarities. For new disease *d*_*t*_, association scores were recalculated by

(15) SM(,*dt)=∑du∈DpSD(dt,du)×SM(,*du)∑du∈DpS⁢D⁢(dt,du)

where *D*_*p*_ represented sets of diseases that have at least one known associations with miRNAs: $D_{p}=\left\{d_{j}|\sum_{i=1}^{m}A\,\left(i,j\right)\neq 0\right\}$. Similarly, in the disease space, *S*_*D*_ was calculated as follows:

(16) $$S_{D}=K\,D\,{\left(K\,D+\lambda \,I\right)}^{-1}\,A^{T}$$

For new miRNA *m*_*t*_, association scores were inferred as follows:

(17) SD(,*mt)=∑mu∈MpSM(mt,mu)×SD(,*mu)∑mu∈MpS⁢M⁢(mt,mu)

The prediction scores in each base learning were obtained as follows:

(18) $$S=0.5\,S_{M}+0.5\,S_{D}^{T}$$

**FIGURE 2:**
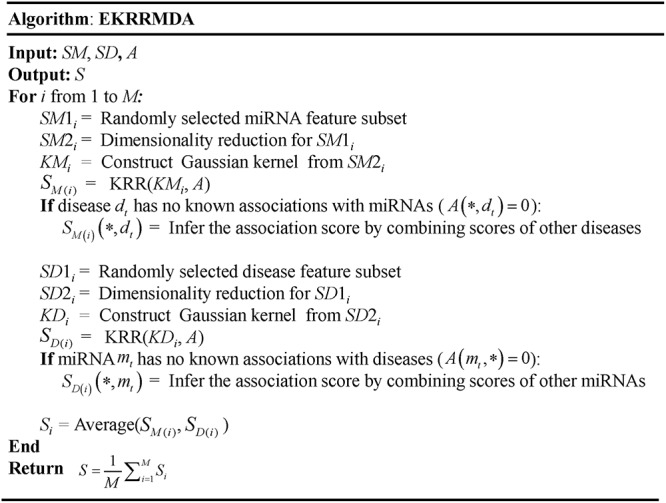
The pseudocode of EKRRMDA.

In base learning, the base classifier that combined two classifiers from miRNA and disease spaces was named as KRR-avg. Above base learning containing three steps was implemented *M* times to yield *M* KRR-avg. The final miRNA-disease association scores could be obtained with average strategy. Figure 2 shows pseudocode of EKRRMDA. For all predicted scores of miRNA-disease pairs with unknown associations, we ranked them and thought that pairs with higher scores were more likely to be associated.

## Results

### Performance Evaluation

In order to assess performance of EKRRMDA, LOOCV and 5-fold cross validation were carried out based on the known miRNA-disease associations from the HMDD V2.0 database (Li et al., 2014), and prediction performance was measured in terms of AUC. Cross validation experiments and AUC measurement were usually used for evaluating methods of miRNA-disease association prediction in many previous important studies (Chen and Huang, 2017; Xiao et al., 2017; You et al., 2017; Chen et al., 2018e; Yang et al., 2018). Particularly, LOOCV was divided into global LOOCV and local LOOCV. In global LOOCV, we selected each known miRNA-disease association as test sample in turn and changed its label “1” to “0” in the adjacency matrix so that the association was hidden. Base on predicted association scores given by EKRRMDA, the test sample was ranked with all miRNA-disease pairs without association evidences. While in local LOOCV, for each given disease *d*, the test sample was one of the miRNAs associated with *d*, and the test sample was ranked with all the unassociated miRNAs for *d*. If the ranking of the test sample exceeded a pre-determined threshold, the model was considered to make a correct prediction for the sample. At different thresholds, the true positive rate (TPR) and the false positive rate (FPR) were calculated to plot the Receiver Operating Characteristic (ROC) curve, where TPR was used as the variate for the vertical axis and FPR for the horizontal axis in ROC. We evaluated the performance of EKRRMDA by calculating the area under ROC curve (AUC).

**FIGURE 3:**
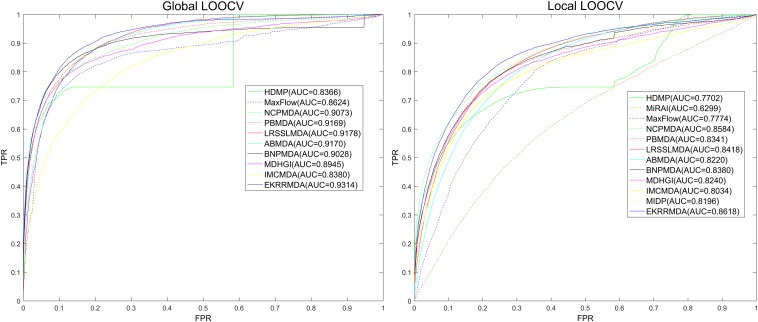
Performance comparisons between EKRRMDA and other 11 prediction models (HDMP, MiRAI, MaxFlow, NCPMDA, PBMDA, LRSSLMDA, ABMDA, BNPMDA, MDHGI, IMCMDA, and MIDP) in terms of ROC curve and AUC based on local and global LOOCV, respectively. As a result, EKRRMDA obtained AUCs of 0.9314 and 0.8618 in the global and local LOOCV, which exceed all the previous classical models.

We compared EKRRMDA with several previous prediction methods in terms of AUC measurement. The overview of these methods was showed in Supplementary Table S1, which briefly provided the characteristic, input data as well as type (Scoring function-based, network-based or machine learning-based) of these models. Figure 3 shows the results of performance comparisons in global and local LOOCV. As a result, EKRRMDA, HDMP, MaxFlow, NCPMDA, PBMDA, LRSSLMDA, ABMDA, BNPMDA, MDHGI, and IMCMDA obtained AUCs of 0.9314, 0.8366, 0.8624, 0.9073, 0.9169, 0.9178, 0.9170, 0.9028, 0.8945, and 0.8380 in global LOOCV, respectively. In local LOOCV, they achieved AUCs of 0.8618, 0.7702, 0.7774, 0.8584, 0.8341, 0.8418, 0.8220, 0.8380, 0.8240, and 0.8034, respectively. In addition, MIDP and MiRAI, obtained AUCs of 0.8196 and 0.6299 in local LOOCV, respectively. MIDP was a local approach that could not predict miRNAs for all diseases simultaneously so that global LOOCV could not evaluate performance of the model. In MiRAI, association scores of samples were closely related to the number of miRNAs associated with the diseases and for a disease with more known associated miRNAs, association scores for its candidate miRNAs tend to be higher. So it was not reasonable to implement prediction for all diseases simultaneously in global LOOCV. Additionally, MiRAI had a low AUC of 0.6299 which was worse than the AUC of 0.867 in Pasquier and Gardes (2016) literature, because MiRAI was a collaborative filtering-based model which was impacted by data sparsity problem. Our training data was HMDD V2.0 containing 383 diseases, where the average number of miRNAs related with a disease was 14, which was sparser than in the dataset in Pasquier and Gardes (2016) study containing 83 diseases with at least 20 known associated miRNAs. From the above comparisons, it is obvious that EKRRMDA has a more reliable performance.

Furthermore, we adopted 5-fold cross validation to evaluate performance of EKRRMDA. At First, we randomly partitioned all known miRNA-disease associations into five equal-sized parts. Each part was taken as the test set in turn, and the remaining four were used for model training. Then, samples in the test set were ranked against the miRNA-disease pairs without known association evidences. Finally, we obtained the rankings of all known associations, and TPR and FPR were calculated at various ranking thresholds to plot ROC and compute AUC. We repeated 5-fold cross-validations 100 times because of random division of known associations. As a result, EKRRMDA, PBMDA, NCPMDA, MaxFlow, HDMP, LRSSLMDA, ABMDA, BNPMDA, MDHGI, and IMCMDA obtained AUCs of 0.9275 ± 0.0008, 0.9127 ± 0.0007, 0.8763 ± 0.0008, 0.8579 ± 0.001, 0.8342 ± 0.0010, 0.9181 ± 0.0004, 0.9023 ± 0.0016, 0.8980 ± 0.0013, 0.8794 ± 0.0021, and 0.8367 ± 0.0005, which further shows the superior performance of our model.

**FIGURE 4:**
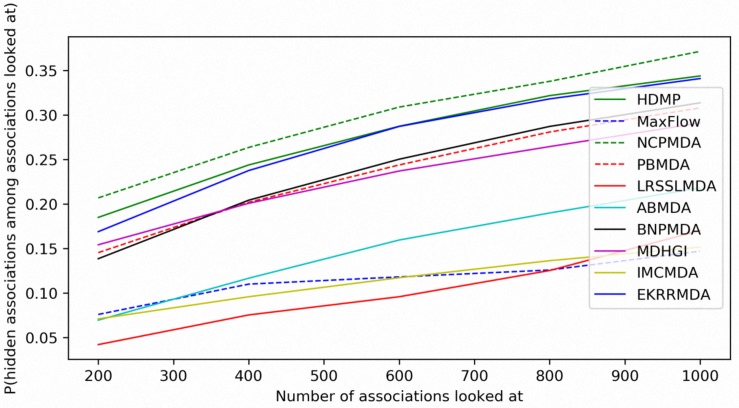
Performance comparisons between EKRRMDA and other prediction models in terms of CDF of ranks based on global LOOCV.

**FIGURE 5:**
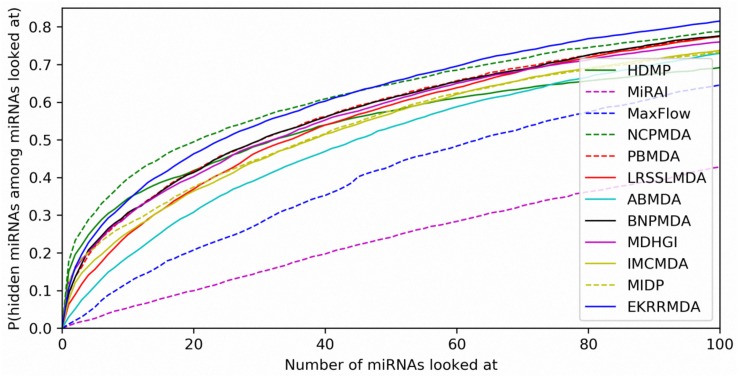
Performance comparisons between EKRRMDA and other prediction models in terms of CDF of ranks based on local LOOCV.

In addition to prediction accuracy, we implemented cumulative distribution function (CDF) for the ranks of predicting samples based on LOOCV results to evaluate the model’s prediction ability, which referred to the (Natarajan and Dhillon, 2014) work on predicting gene-disease associations. Figure 4 showed CDF for the ranks of miRNA-disease associations for different models based on global LOOCV. The vertical axis in the plots gives the probability that a hidden miRNA-disease association is recovered in the top-*k* predictions for various *k* values in the horizontal axis. EKRRMDA outperformed most competitive models under global LOOCV. In the Figure 5, CDF for the miRNA ranks for different models based on local LOOCV was shown. The vertical axis in the plots gives the probability that a hidden miRNA associated with the investigated disease is recovered in the top-*k* predictions for various *k* values in the horizontal axis. EKRRMDA outperformed most competitive models from top 1 to 100 predictions. Specially, the performance of EKRRMDA was weaker than HDMP from top 1 to 10 predictions and NCPMDA from top 1 to 44 predictions, but surpassed HDMP from top 11 to 100 predictions and NCPMDA from top 45 to 100 predictions. However, NCPMDA and HDMP are network-based methods which need reliable similarity measurement for miRNAs and diseases to construct network for prediction. Moreover, it is a significant limitation for HDMP that it could not implement prediction for new diseases having no known association information.

### Model Analysis

In this paper, we constructed prediction model by utilizing random selection of features for ensemble learning, TSVD for feature dimensionality reduction, Gaussian kernel for KRR and average strategy for combining two prediction scores in miRNA and disease space. The section was used to evaluate the effect of these steps.

In our work, KRR-avg as base classifier was constructed by introducing Gaussian kernel which is one of the most popular choices for constructing a kernel from feature vectors. We also compared Gaussian kernel with other two kernel functions used in KRR, Poly(1) and Poly(2) in the literature (Exterkate et al., 2016), both of which are polynomial kernel functions and their corresponding kernel function is κ(*x*,*y*) = 1 + *x*′*y* and κ(*x*,*y*) = (1 + *x*′*y*)^2^, respectively. The results of comparison were shown in Table 1, which indicated that Gaussian kernel performed better than polynomial kernel Poly(1) and Poly(2) in our model.

**TABLE 1:** Comparison of global AUC and local AUC between different kernel functions used in KRR under LOOCV.

**Kernel functions**	**Global AUC**	**Local AUC**
Gaussian kernel	0.9314	0.8618
Poly(1)	0.9163	0.8436
Poly(2)	0.9023	0.8279

We constructed Gaussian kernel for KRR in miRNA and disease space, respectively, and averaged two predictions as final result. An alternative is to combine the kernels into a larger kernel that directly relates miRNA-disease pairs. We employed the Kronecker product kernel to realize it, referring to the literature (Chen and Li, 2017). The Kronecker product *KM*⊗*KD* of the miRNA and disease kernel is

(19) $$K\,\left(\left(m_{i},d_{j}\right),\left(m_{k},d_{l}\right)\right)=K\,M\,\left(m_{i},m_{k}\right)\,K\,D\,\left(d_{j},d_{l}\right)$$

With this kernel, we can implement predictions for all pairs as follows:

(20) $$v\,e\,c\,\left(S^{T}\right)=K\,{\left(K+\sigma \,I\right)}^{-1}\,v\,e\,c\,\left(A^{T}\right)$$

where *vec*(⋅) is the vectorization operator that stacks all columns of a matrix into a column vector. To solve the optimization problem more efficiently, some transformations were made and we can get the prediction in the form of Chen and Li (2017).

(21) $$S=V_{m}\,Z^{T}\,V_{d}^{T}$$

where

$$v\,e\,c\,\left(Z\right)=\left(\Lambda_{m}⊗\Lambda_{d}\right)\,{\left(\Lambda_{m}⊗\Lambda_{d}+\lambda \,I\right)}^{-1}\,v\,e\,c\,\left(V_{d}^{T}\,A^{T}\,V_{m}\right),$$

and _*m*_, _*d*_, *V*_*m*_ and *V*_*d*_ come from the eigen decompositions of the two kernel matrices: $K\,M=V_{m}\,\Lambda_{m}\,V_{m}^{T}$ and $K\,D=V_{d}\,\Lambda_{d}\,V_{d}^{T}$. Under the computational framework of EKRRMDA, we used Kronecker product kernel to combine two Gaussian kernels in a single KRR as base classifier, named Ensemble of Kronecker Kernel Ridge Regression based MiRNA-Disease Association prediction (EKKRRMDA) for this method. The results of comparison between EKRRMDA and EKKRRMDA were shown in Table 2, which showed that the method of constructing separate KRR in miRNA and disease space, respectively, outperformed the method of combining two kernels for a single KRR.

**TABLE 2:** Comparison of global AUC and local AUC between EKRRMDA and EKKRRMDA under LOOCV.

**Methods**	**Global AUC**	**Local AUC**
EKRRMDA	0.9314	0.8618
EKKRRMDA	0.9093	0.8332

Ensemble of Kernel Ridge Regression based MiRNA-Disease Association prediction trained multiple classifiers based on random selection of features, which inevitably brought some noise or redundancy. To address the issue, we implemented dimensionality reduction for the feature subset in each base learning. In addition, dimensionality reduction could reduce computation complexity for each base classifier. In order to evaluate the contribution of random selection of features and dimensionality reduction for EKRRMDA, we implemented three experiments including no random selection of features (i.e., all features were training for one classifier in miRNA and disease space, respectively), no dimensionality reduction and no both (no random selection of features and dimensionality reduction). The comparison results were shown in Table 3, which indicated that both random selection of features and dimensionality reduction could improve prediction performance and especially, random selection of features for ensemble learning contribute more.

**TABLE 3:** Comparison of global AUC and local AUC between EKRRMDA and variants of EKRRMDA under LOOCV.

**Methods**	**Global AUC**	**Local AUC**
EKRRMDA	0.9314	0.8618
No dimensionality reduction	0.9211	0.8568
No random selection of features	0.9015	0.8467
No dimensionality reduction and	0.8951	0.8436
random selection of features		

### Sensitivity Analysis

Here, we made sensitivity analysis for Gaussian kernel parameter, which was vital to the construction of classifiers in our model. The choice of Gaussian kernel parameter is always important but also tricky problem. Some methodologies for optimizing the kernel parameter have been proposed and used in Gaussian kernel methods. Grid search is often used to optimize the Gaussian kernel parameter, which choose the optimal parameter that show best test precision from candidate grid points. The problem of tuning kernel parameter is also done by minimizing an estimate of the generalization error or some other related performance measure (Chapelle et al., 2002; Duan et al., 2003). Moreover, the optimization criterion based on kernel target alignment is a widely used method for choice of Gaussian kernel parameter (Cristianini et al., 2006; Fauvel, 2012).

**FIGURE 6:**
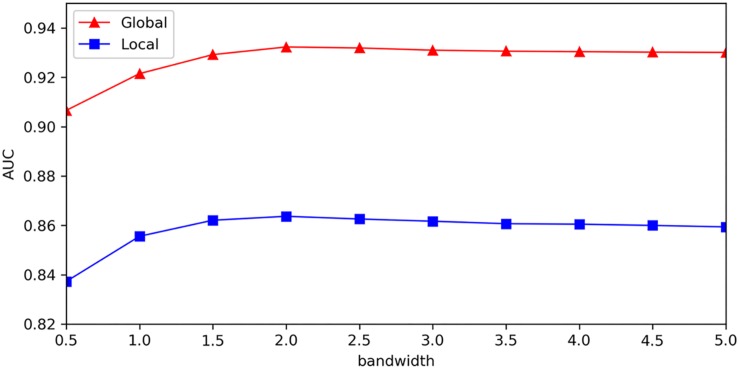
Sensitivity analysis for bandwidth of Gaussian kernel.

We provided sensitivity analysis for Gaussian kernel parameter, which was implemented to investigate the variation of model’s test precision for different parameter values. The results of sensitivity analysis were measured with global AUC and local AUC under the framework of LOOCV, which represented global prediction ability (for all miRNA-disease candidates) and local prediction ability (for miRNA candidates to the investigated disease), respectively. In order to better analyze effect of Gaussian kernel parameter, we adopted other form of Gaussian kernel: $\kappa \,\left(x,y\right)=\exp\left(-\frac{{||x-y||}^{2}}{2\,\sigma^{2}}\right)$ (σis the bandwidth of Gaussian kernel), which was equivalent to $\kappa \,\left(x,y\right)=\exp\left(-\frac{{||x-y||}^{2}}{\gamma }\right)$ used in our model, and made sensitivity analysis for parameter σ. Figure 6 showed that global AUC and local AUC had the same trend and they reached maximum value when σ was about 2.0, and then decreased at a slower rate when increased. As mentioned above, *k*_*m*_ and *k*_*d*_ represented the number of miRNA features and disease features after dimensionality reduction. For Gaussian kernel in our model, we made 2σ^2^equal to *k*_*m*_ for miRNAs and *k*_*d*_ for diseases, so their corresponding σ is 3.1 and 2.7, respectively. From the sensitivity analysis results, we think it is sound to choose Gaussian kernel parameter by setting 2σ^2^ as number of features in our model.

### Case Studies

To demonstrate the prediction accuracy of EKRRMDA, we implemented three different types of case studies on five diseases. The first type of case studies was carried out on three diseases, namely, Esophageal Neoplasms (EN), Kidney Neoplasms (KN) and Lymphoma. The known miRNA-disease associations in HMDD V2.0 were used as the training dataset for our model.

Esophageal Neoplasms, including squamous cell carcinoma (SCC) and adenocarcinoma (ADC), is one of the most common digestive cancers and ranks sixth among all cancers in mortality (Zhang, 2013). It is estimated there are about 15,690 people dying from EN among 16,940 newly diagnosed EN cases in 2017 in the United States (Siegel et al., 2017). Some miRNAs have been confirmed to be closely related to EN in previous studies. For instance, one of latest reports suggested that various miRNAs (miR-144, miR-451, miR-98, miR-10b, and miR-363) were involved in EN by regulating their target genes (Du and Zhang, 2017). In our case study of EN, there were total top 10 and 45 out of the top 50 potential EN-related miRNAs confirmed in dbDEMC and miR2Database (see Table 4).

**TABLE 4:** Prediction of the top 50 predicted miRNAs associated with EN.

**miRNA**	**Evidence**	**miRNA**	**Evidence**
hsa-mir-125b	dbdemc	hsa-mir-29b	dbdemc
hsa-mir-1	dbdemc	hsa-mir-9	dbdemc
hsa-mir-17	dbdemc	hsa-mir-195	dbdemc
hsa-mir-200b	dbdemc	hsa-let-7g	dbdemc
hsa-mir-221	dbdemc	hsa-mir-7	dbdemc
hsa-mir-16	dbdemc	hsa-mir-24	dbdemc
hsa-let-7e	dbdemc	hsa-mir-106a	dbdemc
hsa-mir-222	dbdemc	hsa-mir-106b	dbdemc
hsa-mir-133b	dbdemc	hsa-mir-199b	dbdemc
hsa-mir-30a	dbdemc	hsa-mir-122	unconfirmed
hsa-mir-18a	dbdemc	hsa-mir-124	dbdemc
hsa-mir-142	dbdemc	hsa-mir-30c	dbdemc
hsa-mir-29a	dbdemc	hsa-mir-429	dbdemc
hsa-mir-182	dbdemc	hsa-mir-132	dbdemc
hsa-mir-19b	dbdemc	hsa-mir-15b	dbdemc
hsa-mir-10b	dbdemc	hsa-mir-335	dbdemc
hsa-let-7f	unconfirmed	hsa-mir-224	dbdemc
hsa-let-7d	dbdemc	hsa-mir-127	dbdemc
hsa-mir-146b	dbdemc	hsa-mir-93	dbdemc
hsa-mir-181a	dbdemc	hsa-mir-497	dbdemc
hsa-mir-181b	dbdemc	hsa-mir-137	dbdemc
hsa-mir-125a	dbdemc	hsa-mir-18b	dbdemc
hsa-mir-107	dbdemc; miR2Disease	hsa-mir-204	unconfirmed
hsa-let-7i	dbdemc	hsa-mir-103a	unconfirmed
hsa-mir-218	unconfirmed	hsa-mir-23b	dbdemc

*The first column records top 1–25 related miRNAs. The third column records the top 26–50 related miRNAs.*

Kidney Neoplasms, also known as renal cancer, accounts for about 3% of all adult neoplasms and its incidence rate is also increasing (Gonzalez-Satue et al., 2015). About 87% KN cases in adults were Renal cell carcinoma (RCC) that is the most common malignant epithelial tumor (Mytsyk et al., 2014). Some miRNA-KN associations have been revealed by experimental studies. For instance, a recent study indicated a clear correlation between higher expression of miR-21 and an aggravation in KN, which showed that miR-21 was useful in monitoring KN (Zaman et al., 2012). In addition, multiple miRNAs, including miR-215, miR-200c, miR-192, miR-194, and miR-141 were found downregulated in KN (Senanayake et al., 2012). After implementing EKRRMDA to predict potential KN-related miRNAs, we obtained that 8 miRNAs in top 10 predictions and 43 miRNAs in top 50 predictions were verified by dbDEMC (Yang et al., 2010) and miR2Disease (Jiang et al., 2009) (see Supplementary Table S2).

Lymphoma, a group of blood cell tumors, develops from lymphocytes and includes two main types, namely, Hodgkin Lymphoma and non-Hodgkin Lymphoma (NHL) (Leich et al., 2011). According to research, about 90 percent of the Lymphoma cases are NHL (Alizadeh et al., 2000). There are plenty of miRNAs confirmed to be connected with Lymphoma. For example, miR-155 (contained in the BIC gene) is strongly up-regulated in Burkitt Lymphoma and several other types of Lymphomas (Metzler, 2004). And miRNA hsa-mir-19a exhibited an increased expression level compared with normal canine peripheral blood mononuclear cells (PBMC) and normal lymph nodes (LN) in canine B-cell Lymphomas (Uhl et al., 2011). After implementing EKRRMDA to predict potential lymphoma-related miRNAs we obtained that 8 miRNAs in top10 predictions and 43 miRNAs in top 50 miRNAs were verified by dbDEMC and miR2Disease (see Supplementary Table S3).

The second type of case study on Lung Neoplasms (LN) was implemented based on known associations in HMDD V2.0 database to illustrate the ability of EKRRMDA to predict miRNAs associated with the new disease. We hid all known miRNA-LN associations by changing their labels to “0” in adjacency matrix so that the LN could be treated as a new disease. We obtained a ranking list of miRNA-LN association scores and the top 50 potential miRNAs were shown in Table 5. Verification results showed that 49 miRNAs in top 50 predictions were confirmed by the dbDEMC, miR2Disease, and HMDD V2.0 databases. For example, a study in HMDD V2.0 indicated that expression of miRNA has-mir-21 (ranked first in the top 50 predictions), was more than two times in the squamous cell LN tissues compared with normal tissues (Gao et al., 2011).

**TABLE 5:** Prediction of the top 50 predicted miRNAs associated with LN as a new disease by removing all known associations containing LN in HMDD V2.0 database.

**miRNA**	**Evidence**	**miRNA**	**Evidence**
hsa-mir-21	dbdemc;miR2Disease;HMDD V2.0	hsa-let-7e	miR2Disease;HMDD V2.0
hsa-mir-125b	miR2Disease;HMDD V2.0	hsa-mir-122	unconfirmed
hsa-mir-155	dbdemc;miR2Disease;HMDD V2.0	hsa-let-7f	miR2Disease;HMDD V2.0
hsa-mir-31	dbdemc;miR2Disease;HMDD V2.0	hsa-mir-183	dbdemc;miR2Disease;HMDD V2.0
hsa-mir-375	dbdemc;HMDD V2.0	hsa-mir-148a	dbdemc;HMDD V2.0
hsa-let-7a	dbdemc;miR2Disease;HMDD V2.0	hsa-mir-222	dbdemc;HMDD V2.0
hsa-let-7b	miR2Disease;HMDD V2.0	hsa-mir-200a	dbdemc;miR2Disease;HMDD V2.0
hsa-mir-34c	dbdemc;HMDD V2.0	hsa-mir-199a	dbdemc;miR2Disease;HMDD V2.0
hsa-mir-7	miR2Disease;HMDD V2.0	hsa-mir-214	dbdemc;miR2Disease;HMDD V2.0
hsa-mir-200b	dbdemc; miR2Disease;HMDD V2.0	hsa-mir-1	dbdemc;miR2Disease;HMDD V2.0
hsa-mir-15b	dbdemc	hsa-mir-221	dbdemc;HMDD V2.0
hsa-mir-16	dbdemc;miR2Disease	hsa-mir-133a	dbdemc;HMDD V2.0
hsa-mir-34a	dbdemc;HMDD V2.0	hsa-mir-218	dbdemc;miR2Disease;HMDD V2.0
hsa-let-7g	dbdemc; miR2Disease; HMDD V2.0	hsa-mir-146a	dbdemc;miR2Disease;HMDD V2.0
hsa-let-7i	dbdemc;HMDD V2.0	hsa-mir-26a	dbdemc;miR2Disease;HMDD V2.0
hsa-let-7c	dbdemc; miR2Disease; HMDD V2.0	hsa-mir-205	dbdemc;miR2Disease;HMDD V2.0
hsa-mir-196a	dbdemc;HMDD V2.0	hsa-mir-206	HMDD V2.0
hsa-mir-141	dbdemc;miR2Disease	hsa-mir-19a	dbdemc;miR2Disease;HMDD V2.0
hsa-mir-34b	dbdemc;HMDD V2.0	hsa-mir-200c	dbdemc;miR2Disease;HMDD V2.0
hsa-mir-100	dbdemc;HMDD V2.0	hsa-mir-133b	dbdemc;miR2Disease;HMDD V2.0
hsa-let-7d	dbdemc;miR2Disease;HMDD V2.0	hsa-mir-29c	dbdemc;miR2Disease;HMDD V2.0
hsa-mir-203	dbdemc;miR2Disease;HMDD V2.0	hsa-mir-106b	dbdemc
hsa-mir-9	miR2Disease;HMDD V2.0	hsa-mir-93	dbdemc;miR2Disease;HMDD V2.0
hsa-mir-145	dbdemc;miR2Disease;HMDD V2.0	hsa-mir-15a	dbdemc
hsa-mir-101	dbdemc;miR2Disease;HMDD V2.0	hsa-mir-499a	HMDD V2.0

*The first column records top 1–25 related miRNAs. The third column records the top 26–50 related miRNAs.*

Finally, to evaluate performance of EKRRMDA on different dataset, we implemented the third type of case study on Breast Neoplasms (BN) based on the known associations in HMDD V1.0 database that covers 1395 known miRNA-disease associations between 271 miRNAs and 137 diseases. Respectively, 10, 20, and 48 miRNAs in top 10, 20, 50 predictions were confirmed by dbDEMC, miR2Disease, and HMDD V2.0 (see Table 6). For example, has-let-7e, the miRNA ranked first in the top 50 predictions, was found to have close relationship with the development of BN in Chinese women (Jiang et al., 2013).

**TABLE 6:** Prediction of the top 50 predicted miRNAs associated with BN based on known associations in HMDD V1.0 database.

**miRNA**	**Evidence**	**miRNA**	**Evidence**
hsa-let-7e	dbdemc;HMDD V2.0	hsa-mir-195	dbdemc;miR2Disease;HMDD V2.0
hsa-let-7b	dbdemc;HMDD	hsa-mir-196b	dbdemc
hsa-let-7i	dbdemc;miR2Disease;HMDD V2.0	hsa-mir-203	dbdemc;miR2Disease;HMDD V2.0
hsa-mir-223	dbdemc;HMDD V2.0	hsa-mir-142	unconfirmed
hsa-let-7c	dbdemc;HMDD V2.0	hsa-mir-30e	unconfirmed
hsa-let-7g	dbdemc;HMDD V2.0	hsa-mir-32	dbdemc
hsa-mir-16	dbdemc;HMDD V2.0	hsa-mir-199b	dbdemc;HMDD V2.0
hsa-mir-126	dbdemc;miR2Disease;HMDD V2.0	hsa-mir-99a	dbdemc
hsa-mir-92a	HMDD V2.0	hsa-mir-23b	dbdemc;HMDD V2.0
hsa-mir-92b	dbdemc	hsa-mir-30a	miR2Disease;HMDD V2.0
hsa-mir-106a	dbdemc	hsa-mir-335	dbdemc;miR2Disease;HMDD V2.0
hsa-mir-101	dbdemc;miR2Disease;HMDD V2.0	hsa-mir-532	dbdemc
hsa-mir-29c	dbdemc;miR2Disease;HMDD V2.0	hsa-mir-107	dbdemc;HMDD V2.0
hsa-mir-191	dbdemc;miR2Disease;HMDD V2.0	hsa-mir-224	dbdemc;HMDD V2.0
hsa-mir-373	dbdemc;miR2Disease;HMDD V2.0	hsa-mir-98	dbdemc;miR2Disease
hsa-mir-99b	dbdemc	hsa-mir-27a	dbdemc;miR2Disease;HMDD V2.0
hsa-mir-182	dbdemc;miR2Disease;HMDD V2.0	hsa-mir-95	dbdemc
hsa-mir-181a	dbdemc;miR2Disease;HMDD V2.0	hsa-mir-128b	miR2Disease
hsa-mir-24	dbdemc;HMDD V2.0	hsa-mir-198	dbdemc
hsa-mir-100	dbdemc;HMDD V2.0	hsa-mir-31	dbdemc;miR2Disease;HMDD V2.0
hsa-mir-15b	dbdemc	hsa-mir-491	dbdemc
hsa-mir-150	dbdemc	hsa-mir-193b	dbdemc;miR2Disease;HMDD V2.0
hsa-mir-18b	dbdemc;HMDD V2.0	hsa-mir-181d	dbdemc;miR2Disease
hsa-mir-372	dbdemc	hsa-mir-183	dbdemc;HMDD V2.0
hsa-mir-130a	dbdemc	hsa-mir-135a	dbdemc;HMDD V2.0

*The first column records top 1–25 related miRNAs. The third column records the top 26–50 related miRNAs.*

In addition, in order to further assess robustness of the model, we introduced random noise by randomly removing 20% known miRNA-disease associations in several case studies, i.e., we randomly changed 20% label “1” to “0” in adjacent matrix. To reduce the bias from random change, we repeated above experiment 10 times. We compared its average performance in top 10 and 50 predictions with our model in case studies. From the Table 7, we can observe that the number of confirmed miRNAs in top 10 and 50 predictions scarcely changed when random noise was introduced into case studies, which could show robustness of the model. To conclude, the case studies discussed above have demonstrated the outstanding prediction accuracy of EKRRMDA. In each case study, most of miRNAs in top 50 predictions were validated to be associate with the investigated disease, and we would expect most of the remaining predictions to be verified in the future.

**TABLE 7:** The number of validated miRNAs among top 10 and top 50 predicted miRNAs in case studies between with all known miRNA-disease associations and with removing 20% associations.

**Case study**	**Top 10 and all**	**Top 50 and all**
	**associations vs.**	**associations vs.**
	**removing 20%**	**removing 20%**
	**associations**	**associations**
The first type and EN	10 vs. 10	45 vs. 47
The first type and KN	8 vs. 8.7	43 vs. 41.6
The first type and Lymphoma	8 vs. 8.5	43 vs. 43.7
The second type and LN	10 vs. 10	49 vs. 48.9
The third type and BN	10 vs. 9.9	48 vs. 48

## Discussion

Considering that it costs much time and money to discover more potential miRNA-disease associations by traditional biological experiments, many computational models were developed to predict potential miRNA-disease associations, which could reduce cost and improve efficiency by preferentially verifying those promising associations. In this paper, we presented a machine-based prediction model named EKRRMDA. The novelty of the model was 2-fold. The first novelty was computational framework based on ensemble learning and feature dimensionality reduction. Since ensemble learning has been widely used to improve prediction accuracy, it was also worthwhile to design an ensemble learning model for prediction of potential miRNA-disease associations. In our prediction model, multiple base learnings were constructed based on random miRNA (disease) feature selection, each of which generated corresponding base classifier. However, our proposed ensemble learning model would increase computation complexity and inevitably brought some noise or redundancy, which motivated us employ feature dimensionality reduce techniques to address these issues. The second novelty was base classifier of the model. In this paper, we chose KRR as base classifier that always have been applied to drug-target association prediction and achieved excellent results (van Laarhoven and Marchiori, 2013), but to our knowledge, it has not been used for miRNA-disease association prediction. In model evaluation, Cross validations and case studies on EN, KN, Lymphoma, LN, and BN have shown the outstanding performance of EKRRMDA. We conclude that EKRRMDA would be a reliable computational model to predict disease-related miRNAs and could provide a substantial help in the prevention, diagnosis and treatment of human diseases.

The prominent performance of the model could be attributed to the following points. Firstly, for the base classifier, we established a bipartite local model by constructing two classifiers with KRR in two different spaces (the miRNA space and the disease space), which could solve the problem encountered by previous methods in figuring out a suitable way to merge miRNA and disease information. Secondly, multiple base classifiers were trained and integrated with ensemble learning strategy which generally bring more prediction accuracy than single classifier. Thirdly, we used the dimension reduction technique to eliminate noises, redundancy, or irrelevant information in the computation, which not only decreased the computational complexity, but also improved the prediction accuracy of the model.

However, the method had several limitations. First, the current known miRNA-disease associations were still inadequate for making much mre accuracy predictions, and with the increase of biological data in the future, the prediction performance of this method could be further improved. Second, similarity calculations for miRNAs and diseases had important impact on performance of model. We believe that integrating more biological information would contribute to obtaining more reliable similarity measures. Third, the choice of the parameter values remained to be further studied, such as parameter *r* used in random feature selection, truncation parameter *k*_*m*_ used in feature dimensionality reduction with TSVD. Especially, how to reasonably integrate results from different spaces would be a critical problem for future research.

## Data Availability Statement

All datasets generated for this study are included in the article/Supplementary Material.

## Author Contributions

L-HP implemented the experiments, analyzed the result, and wrote the manuscript. L-QZ conceived the project, analyzed the result, and revised the manuscript. XC conceived the project, developed the prediction method, designed the experiments, analyzed the result, and revised the manuscript. XP analyzed the result and revised the manuscript. All authors read and approved the final manuscript.

## Conflict of Interest

The authors declare that the research was conducted in the absence of any commercial or financial relationships that could be construed as a potential conflict of interest.

## Footnotes

1 http://www.nlm.nih.gov/

## References

[B1] Alizadeh A. A. Eisen M. B. Davis R. E. Ma C. Lossos I. S. Rosenwald A. (2000). Distinct types of diffuse large B-cell lymphoma identified by gene expression profiling. *Nature* 403 503–511. 10.1038/35000501 10676951

[B2] Bang C. Fiedler J. Thum T. (2012). Cardiovascular importance of the microRNA-23/27/24 family. *Microcirculation* 19 208–214. 10.1111/j.1549-8719.2011.00153.x 22136461

[B3] Bartel D. P. (2004). MicroRNAs: genomics, biogenesis, mechanism, and function. *Cell* 116 281–297. 14744438 10.1016/s0092-8674(04)00045-5

[B4] Bruce J. P. Hui A. B. Y. Shi W. Perezordonez B. Weinreb I. Xu W. (2015). Identification of a microRNA signature associated with risk of distant metastasis in nasopharyngeal carcinoma. *Oncotarget* 6 4537–4550. 25738365 10.18632/oncotarget.3005 PMC4414210

[B5] Calin G. A. Croce C. M. (2006). MicroRNA signatures in human cancers. *Nat. Rev. Cancer* 6 857–866. 10.1038/nrc1997 17060945

[B6] Chapelle O. Vapnik V. Bousquet O. Mukherjee S. (2002). Choosing Multiple Parameters for Support Vector Machines. *Mach. Learn.* 46 131–159. 10.1023/a:1012450327387

[B7] Chen H. Li J. (2017). “A Flexible and Robust Multi-Source Learning Algorithm for Drug Repositioning,” in *Proceedings of the 8th ACM International Conference on Bioinformatics, Computational Biology, and Health Informatics*, (Boston, MA), 510–515.

[B8] Chen X. Cheng J. Y. Yin J. (2018a). Predicting microRNA-disease associations using bipartite local models and hubness-aware regression. *RNA Biol.* 15 1192–1205. 10.1080/15476286.2018.1517010 30196756 PMC6284580

[B9] Chen X. Guan N. N. Sun Y. Z. Li J. Q. Qu J. (2018b). MicroRNA-small molecule association identification: from experimental results to computational models. *Brief. Bioinform.* bby098. 10.1093/bib/bby098 30325405

[B10] Chen X. Huang L. (2017). LRSSLMDA: laplacian regularized sparse subspace learning for MiRNA-disease association prediction. *PLoS Comput. Biol.* 13:e1005912. 10.1371/journal.pcbi.1005912 29253885 PMC5749861

[B11] Chen X. Huang L. Xie D. Zhao Q. (2018c). EGBMMDA: extreme gradient boosting machine for MiRNA-disease association prediction. *Cell Death Dis.* 9 3. 10.1038/s41419-017-0003-x 29305594 PMC5849212

[B12] Chen X. Jiang Z. C. Xie D. Huang D. S. Zhao Q. Yan G. Y. (2017a). A novel computational model based on super-disease and miRNA for potential miRNA-disease association prediction. *Mol. Biosyst.* 13 1202–1212. 10.1039/c6mb00853d 28470244

[B13] Chen X. Liu M. X. Yan G. Y. (2012). RWRMDA: predicting novel human microRNA-disease associations. *Mol. Biosyst.* 8 2792–2798. 10.1039/c2mb25180a 22875290

[B14] Chen X. Ren B. Chen M. Wang Q. Zhang L. Yan G. (2016a). NLLSS: predicting synergistic drug combinations based on semi-supervised learning. *PLoS Comput. Biol.* 12:e1004975. 10.1371/journal.pcbi.1004975 27415801 PMC4945015

[B15] Chen X. Wang L. Qu J. Guan N.-N. Li J.-Q. (2018d). Predicting miRNA–disease association based on inductive matrix completion. *Bioinformatics* 34 4256–4265. 29939227 10.1093/bioinformatics/bty503

[B16] Chen X. Wu Q. F. Yan G. Y. (2017b). RKNNMDA: ranking-based KNN for MiRNA-disease association prediction. *RNA Biol.* 14 952–962. 10.1080/15476286.2017.1312226 28421868 PMC5546566

[B17] Chen X. Xie D. Wang L. Zhao Q. You Z. H. Liu H. (2018e). BNPMDA: Bipartite network projection for MiRNA-disease association prediction. *Bioinformatics* 34 3178–3186. 10.1093/bioinformatics/bty333 29701758

[B18] Chen X. Xie D. Zhao Q. You Z. H. (2019). MicroRNAs and complex diseases: from experimental results to computational models. *Briefings Bioinform.* 20 515–539. 10.1093/bib/bbx130 29045685

[B19] Chen X. Yan G. Y. (2014). Semi-supervised learning for potential human microRNA-disease associations inference. *Sci. Rep.* 4:5501. 10.1038/srep05501 24975600 PMC4074792

[B20] Chen X. Yan C. C. Zhang X. You Z. H. (2017c). Long non-coding RNAs and complex diseases: from experimental results to computational models. *Briefings Bioinform.* 18 558–576. 10.1093/bib/bbw060 27345524 PMC5862301

[B21] Chen X. Yan C. C. Zhang X. Li Z. Deng L. Zhang Y. (2015). RBMMMDA: predicting multiple types of disease-microRNA associations. *Sci. Rep.* 5:13877. 10.1038/srep13877 26347258 PMC4561957

[B22] Chen X. Yan C. C. Zhang X. You Z. H. Deng L. Liu Y. (2016b). WBSMDA: within and between score for MiRNA-disease association prediction. *Sci. Rep.* 6:21106. 10.1038/srep21106 26880032 PMC4754743

[B23] Chen X. Yan C. C. Zhang X. You Z. H. Huang Y. A. Yan G. Y. (2016c). HGIMDA: heterogeneous graph inference for miRNA-disease association prediction. *Oncotarget* 7 65257–65269. 10.18632/oncotarget.11251 27533456 PMC5323153

[B24] Chen X. Yan C. C. Zhang X. Zhang X. Dai F. Yin J. (2016d). Drug-target interaction prediction: databases, web servers and computational models. *Briefings Bioinform.* 17 696–712. 10.1093/bib/bbv066 26283676

[B25] Chen X. Yin J. Qu J. Huang L. (2018f). MDHGI: matrix decomposition and heterogeneous graph inference for miRNA-disease association prediction. *PLoS Comput. Biol.* 14:e1006418. 10.1371/journal.pcbi.1006418 30142158 PMC6126877

[B26] Chen X. Zhou Z. Zhao Y. (2018g). ELLPMDA: Ensemble learning and link prediction for miRNA-disease association prediction. *RNA Biol.* 15 807–818. 10.1080/15476286.2018.1460016 29619882 PMC6152467

[B27] Cristianini N. Kandola J. Elisseeff A. Shawe-Taylor J. (2006). “On Kernel Target Alignment,” in *Innovations in Machine Learning: Theory and Applications*. Berlin, Heidelberg, eds Holmes D. E. Jain L. C., (Berlin: Springer), 205–256. 10.1007/10985687_8

[B28] Du J. Zhang L. (2017). Analysis of salivary microRNA expression profiles and identification of novel biomarkers in esophageal cancer. *Oncol. Lett.* 14 1387–1394. 10.3892/ol.2017.6328 28789354 PMC5529882

[B29] Duan K. Keerthi S. S. Poo A. N. (2003). Evaluation of simple performance measures for tuning SVM hyperparameters. *Neurocomputing* 51 41–59. 10.1016/S0925-2312(02)00601-X

[B30] Esquelakerscher A. Slack F. J. (2006). Oncomirs - microRNAs with a role in cancer. *Nat. Rev. Cancer* 6:259. 10.1038/nrc1840 16557279

[B31] Exterkate P. Groenen P. J. F. Heij C. van Dijk D. (2016). Nonlinear forecasting with many predictors using kernel ridge regression. *Int. J. Forecasting* 32 736–753. 10.1016/j.ijforecast.2015.11.017

[B32] Ezzat A. Wu M. Li X. L. Kwoh C. K. (2017). Drug-target interaction prediction using ensemble learning and dimensionality reduction. *Methods* 129 81–88. 10.1016/j.ymeth.2017.05.016 28549952

[B33] Fauvel M. (ed.) (2012). “Kernel matrix approximation for learning the kernel hyperparameters,” in *2012 IEEE International Geoscience and Remote Sensing Symposium*, (Munich).

[B34] Gao W. Shen H. Liu L. Xu J. Xu J. Shu Y. (2011). MiR-21 overexpression in human primary squamous cell lung carcinoma is associated with poor patient prognosis. *J. Cancer Res. Clin. Oncol.* 137 557–566. 10.1007/s00432-010-0918-4 20508945 PMC11828261

[B35] Gonzalez-Satue C. Canas-Sole L. Valverde-Vilamala I. Tapia-Garcia M. Pereira-Barrios J. C. Areal-Calama J. (2015). Neoplasm in kidney graft. *Anal. Cause Tumour Treatm. Opt.* 35 87–91. 10.3265/Nefrologia.pre2014.Sep.12739 25611837

[B36] Gu C. Liao B. Li X. Li K. (2016). Network consistency projection for human miRNA-disease associations inference. *Sci. Rep.* 6:36054. 10.1038/srep36054 27779232 PMC5078764

[B37] Jeong H. C. Kim E. K. Lee J. H. Lee J. M. Yoo H. N. Kim J. K. (2011). Aberrant expression of let-7a miRNA in the blood of non-small cell lung cancer patients. *Mol. Med. Rep.* 4 383–387. 10.3892/mmr.2011.430 21468581

[B38] Jiang Q. Wang Y. Hao Y. Juan L. Teng M. Zhang X. (2009). miR2Disease: a manually curated database for microRNA deregulation in human disease. *Nucleic Acids Res.* 37 D98–D104. 10.1093/nar/gkn714 18927107 PMC2686559

[B39] Jiang Y. Qin Z. Hu Z. Guan X. Wang Y. He Y. (2013). Genetic variation in a hsa-let-7 binding site in RAD52 is associated with breast cancer susceptibility. *Carcinogenesis* 34 689–693. 10.1093/carcin/bgs373 23188672

[B40] Jiang Q. Wang G. Zhang T. Wang Y. (2010). Predicting human microRNA-disease associations based on support vector machine. . *IEEE* 467–472. 10.1109/BIBM.2010.5706611 24417022

[B41] Jopling C. L. Yi M. Lancaster A. M. Lemon S. M. Sarnow P. (2005). Modulation of hepatitis C virus RNA abundance by a liver-specific MicroRNA. *Science* 309 1577–1581. 10.1126/science.1113329 16141076

[B42] Latronico M. V. Catalucci D. Condorelli G. (2007). Emerging role of microRNAs in cardiovascular biology. *Circ. Res.* 101 1225–1236. 10.1161/circresaha.107.163147 18063818

[B43] Leich E. Zamo A. Horn H. Haralambieva E. Puppe B. Gascoyne R. D. (2011). MicroRNA profiles of t(14;18)-negative follicular lymphoma support a late germinal center B-cell phenotype. *Blood* 118 5550–5558. 10.1182/blood-2011-06-361972 21960592 PMC3217356

[B44] Li J. Q. Rong Z. H. Chen X. Yan G. Y. You Z. H. (2017). MCMDA: matrix completion for MiRNA-disease association prediction. *Oncotarget* 8 21187–21199. 10.18632/oncotarget.15061 28177900 PMC5400576

[B45] Li Y. Qiu C. Tu J. Geng B. Yang J. Jiang T. (2014). HMDD v2.0: a database for experimentally supported human microRNA and disease associations. *Nucleic Acids Res.* 42 D1070. 10.1093/nar/gkt1023 24194601 PMC3964961

[B46] Liang P. Lv C. Jiang B. Long X. Zhang P. Zhang M. (2012). MicroRNA profiling in denatured dermis of deep burn patients. *Burns* 38 534–540. 10.1016/j.burns.2011.10.014 22360957

[B47] Lipscomb C. E. (2000). Medical subject headings (MeSH). *Bull. Med. Library Assoc.* 88 265–266. PMC35238 10928714

[B48] Lynam−Lennon N. Maher S. G. Reynolds J. V. (2009). The roles of microRNA in cancer and apoptosis. *Biol. Rev.* 84 55–71. 10.1111/j.1469-185x.2008.00061.x 19046400

[B49] Meola N. Gennarino V. A. Banfi S. (2009). microRNAs and genetic diseases. *Pathogenetics* 2:7. 10.1186/1755-8417-2-7 19889204 PMC2778645

[B50] Metzler M. (2004). High expression of precursor microRNA-155/BIC RNA in children with Burkitt lymphoma. *Genes Chromosomes Cancer* 39 167–169. 10.1002/gcc.10316 14695998

[B51] Milanese J. S. Tibiche C. Zou J. Meng Z. Nantel A. Drouin S. (2019). Germline variants associated with leukocyte genes predict tumor recurrence in breast cancer patients. *NPJ Precis. Oncol.* 3:28. 10.1038/s41698-019-0100-7 31701019 PMC6825127

[B52] Mørk S. Pletscherfrankild S. Palleja C. A. Gorodkin J. Jensen L. J. (2014). Protein-driven inference of miRNA-disease associations. *Bioinformatics* 30:392. 10.1093/bioinformatics/btt677 24273243 PMC3904518

[B53] Mytsyk Y. Borys Y. Komnatska I. Dutka I. Shatynskamytsyk I. (2014). Value of the diffusion-weighted MRI in the differential diagnostics of malignant and benign kidney neoplasms – our clinical experience. *Polish J. Radiol.* 79 290–295. 10.12659/PJR.890604 25202435 PMC4156335

[B54] Natarajan N. Dhillon I. S. (2014). Inductive matrix completion for predicting gene-disease associations. *Bioinformatics* 30 i60–i68. 10.1093/bioinformatics/btu269 24932006 PMC4058925

[B55] Pasquier C. Gardes J. (2016). Prediction of miRNA-disease associations with a vector space model. *Sci. Rep.* 6:27036. 10.1038/srep27036 27246786 PMC4887905

[B56] Reddy K. B. (2015). MicroRNA (miRNA) in cancer. *Cancer Cell Int.* 15 1–6. 25960691 10.1186/s12935-015-0185-1 PMC4424445

[B57] Rojano E. Seoane P. Ranea J. A. G. Perkins J. R. (2019). Regulatory variants: from detection to predicting impact. *Briefings Bioinform.* 20 1639–1654. 10.1093/bib/bby039 29893792 PMC6917219

[B58] Senanayake U. Das S. Vesely P. Alzoughbi W. Frohlich L. F. Chowdhury P. (2012). miR-192, miR-194, miR-215, miR-200c and miR-141 are downregulated and their common target ACVR2B is strongly expressed in renal childhood neoplasms. *Carcinogenesis* 33 1014–1021. 10.1093/carcin/bgs126 22431721

[B59] Shi H. Xu J. Zhang G. Xu L. Li C. Wang L. (2013). Walking the interactome to identify human miRNA-disease associations through the functional link between miRNA targets and disease genes. *Bmc Syst. Biol.* 7:101. 10.1186/1752-0509-7-101 24103777 PMC4124764

[B60] Siegel R. L. Miller K. D. Jemal A. (2017). Cancer statistics, 2017. *Cancer J. Clin.* 67 7–30. 10.3322/caac.21387 28055103

[B61] Uhl E. Krimer P. Schliekelman P. Tompkins S. M. Suter S. (2011). Identification of altered MicroRNA expression in canine lymphoid cell lines and cases of B- and T-Cell lymphomas. *Genes Chromosomes Cancer* 50 950–967. 10.1002/gcc.20917 21910161

[B62] van Laarhoven T. Marchiori E. (2013). Predicting drug-target interactions for new drug compounds using a weighted nearest neighbor profile. *PLoS One* 8:e66952. 10.1371/journal.pone.0066952 23840562 PMC3694117

[B63] van Laarhoven T. Nabuurs S. B. Marchiori E. (2011). Gaussian interaction profile kernels for predicting drug-target interaction. *Bioinformatics* 27 3036–3043. 10.1093/bioinformatics/btr500 21893517

[B64] Vasudevan S. Tong Y. Steitz J. A. (2007). Switching from repression to activation: microRNAs can up-regulate translation. *Science* 318 1931–1934. 10.1126/science.1149460 18048652

[B65] Vovk V. (2013). *Kernel Ridge Regression.* Berlin: Springer, 105–116.

[B66] Wang C. C. Chen X. Yin J. Qu J. (2019). An integrated framework for the identification of potential miRNA-disease association based on novel negative samples extraction strategy. *RNA Biol.* 16 257–269. 10.1080/15476286.2019.1568820 30646823 PMC6380288

[B67] Wang D. Wang J. Lu M. Song F. Cui Q. (2010). Inferring the human microRNA functional similarity and functional network based on microRNA-associated diseases. *Bioinformatics* 26 1644–1650. 10.1093/bioinformatics/btq241 20439255

[B68] Wilczynska A. Bushell M. (2015). The complexity of miRNA-mediated repression. *Cell Death Differ.* 22 22–33. 10.1038/cdd.2014.112 25190144 PMC4262769

[B69] Xiao Q. Luo J. Liang C. Cai J. Ding P. (2017). A graph regularized non-negative matrix factorization method for identifying microRNA-disease associations. *Bioinformatics* 34 239–248. 10.1093/bioinformatics/btx545 28968779

[B70] Xu J. Li C. X. Lv J. Y. Li Y. S. Xiao Y. Shao T. T. (2011). Prioritizing candidate disease miRNAs by topological features in the miRNA target-dysregulated network: case study of prostate cancer. *Mol. Cancer Ther.* 10:1857. 10.1158/1535-7163.MCT-11-0055 21768329

[B71] Xu P. (1998). Truncated SVD methods for discrete linear ill-posed problems. *Geophys. J. Int.* 135 505–514. 10.1046/j.1365-246X.1998.00652.x

[B72] Xu T. Zheng X. Li B. Jin P. Qin Z. Wu H. (2018). A comprehensive review of computational prediction of genome-wide features. *Briefings Bioinform.* 10.1093/bib/bby110 [Epub ahead of print]. 30462144 PMC10233247

[B73] Xu X. Li J. Zou J. Feng X. Zhang C. Zheng R. (2019). Association of germline variants in natural killer cells with tumor immune microenvironment subtypes, tumor-infiltrating lymphocytes, immunotherapy response, clinical outcomes, and cancer Risk. *JAMA Netw. Open* 2:e199292. 10.1001/jamanetworkopen.2019.9292 31483464 PMC6727785

[B74] Xuan P. Han K. Guo M. Guo Y. Li J. Ding J. (2013). Prediction of microRNAs associated with human diseases based on weighted k most similar neighbors. *Plos One* 8:e70204. 10.1371/journal.pone.0070204 23950912 PMC3738541

[B75] Xuan P. Han K. Guo Y. Li J. Li X. Zhong Y. (2015). Prediction of potential disease-associated microRNAs based on random walk. *Bioinformatics* 31 1805–1815. 10.1093/bioinformatics/btv039 25618864

[B76] Yang Y. Fu X. Qu W. Xiao Y. Shen H.-B. (2018). MiRGOFS: a GO-based functional similarity measurement for miRNAs, with applications to the prediction of miRNA subcellular localization and miRNA–disease association. *Bioinformatics* 34 3547–3556. 10.1093/bioinformatics/bty343 29718114

[B77] Yang Z. Ren F. Liu C. He S. Sun G. Gao Q. (2010). dbDEMC: a database of differentially expressed miRNAs in human cancers. *Bmc Genomics* 11(Suppl. 4):s5. 10.1186/1471-2164-11-s4-s5 21143814 PMC3005935

[B78] You Z. H. Huang Z. A. Zhu Z. Yan G. Y. Li Z. W. Wen Z. (2017). PBMDA: a novel and effective path-based computational model for miRNA-disease association prediction. *PLoS Comput. Biol.* 13:e1005455. 10.1371/journal.pcbi.1005455 28339468 PMC5384769

[B79] Yu H. Chen X. Lu L. (2017). Large-scale prediction of microRNA-disease associations by combinatorial prioritization algorithm. *Sci. Rep.* 7:43792. 10.1038/srep43792 28317855 PMC5357838

[B80] Zaman M. S. Shahryari V. Deng G. Thamminana S. Saini S. Majid S. (2012). Up-regulation of microRNA-21 correlates with lower kidney cancer survival. *PLoS One* 7:e31060. 10.1371/journal.pone.0031060 22347428 PMC3275568

[B81] Zhang Y. (2013). Epidemiology of esophageal cancer. *World J. Gastroenterol.* 19 5598–5606. 10.3748/wjg.v19.i34.5598 24039351 PMC3769895

[B82] Zhao Y. Chen X. Yin J. (2019). Adaptive boosting-based computational model for predicting potential miRNA-disease associations. *Bioinformatics* 35 4730–4738. 10.1093/bioinformatics/btz297 31038664

