# Supplementary material for: A Computational Study of Potential miRNA-Disease Association Inference Based on Ensemble Learning and Kernel Ridge Regression
Source: Front Bioeng Biotechnol. 2020 Feb 6;8:40. doi: 10.3389/fbioe.2020.00040 (PMC7015868; doi:10.3389/fbioe.2020.00040)
Supplement: Supplementary file 1 [file Data_Sheet_1.zip › Supplementary table legends.DOCX]

**A computational study of potential miRNA-disease association inference based on ensemble learning and kernel ridge regression**

Li-Hong Peng^1^, Li-Qian Zhou^1,*^, Xing Chen^2,*^, Xue Piao^3,*^

^1^School of Computer Science, Hunan University of Technology, Zhuzhou, China

^2^School of Information and Control Engineering, China University of Mining and Technology, Xuzhou, China

^3^ School of Medical Informatics, Xuzhou Medical University, Xuzhou, China

**Supplementary Table 1.** We briefly showed the characteristic, input data and type (network-based, scoring function-based and machine learning-based) of EKRRMDA and other 11 previous models.

**Supplementary Table 2.** Prediction of the top 50 predicted miRNAs associated with KN. The first column records top 1-25 related miRNAs. The third column records the top 26-50 related miRNAs.

**Supplementary Table 3.** Prediction of the top 50 predicted miRNAs associated with Lymphoma. The first column records top 1-25 related miRNAs. The third column records the top 26-50 related miRNAs.
